# Lack of specialist nidicoles as a characteristic of mite assemblages inhabiting nests of the ground-nesting wood warbler, *Phylloscopus sibilatrix* (Aves: Passeriformes)

**DOI:** 10.1007/s10493-021-00620-8

**Published:** 2021-05-03

**Authors:** Agnieszka Napierała, Marta Maziarz, Grzegorz Hebda, Richard K. Broughton, Tomasz Rutkowski, Michał Zacharyasiewicz, Jerzy Błoszyk

**Affiliations:** 1 Department of General Zoology, Faculty of Biology Adam, Mickiewicz University, Uniwersytetu Poznańskiego 6, 61-614 Poznań, Poland; 2 Museum and Institute of Zoology, Polish Academy of Sciences, Wilcza 64, 00-679 Warsaw, Poland; 3 Institute of Biology, Opole University, Oleska 22, 45-040 Opole, Poland; 4 UK Centre for Ecology & Hydrology, Crowmarsh Gifford, Maclean Building, Benson Lane, Wallingford, OX10 8BB UK; 5 Faculty of Biology at Adam, Natural History Collections, Mickiewicz University, Uniwersytetu Poznańskiego 6, 61-614 Poznań, Poland

**Keywords:** Assemblage structure, Crotonioidea, Nest of birds, Unstable microhabitats, Uropodina

## Abstract

Bird and mammal nests provide microhabitats that support a range of other species, including invertebrates. However, the variation between communities of nest-dwelling invertebrates in different nests is poorly understood. The major aim of this study was to analyze the assemblage structure of mites from the suborder Uropodina (Acari: Mesostigmata) and from superfamily Crotonioidea (Acari: Oribatida) inhabiting nests of the wood warbler, *Phylloscopus sibilatrix* (Aves: Passeriformes), located on a forest floor in Białowieża Forest, in eastern Poland. We also assessed the correlation between the nest material used by the birds with the assemblage structure of Uropodina mites, and compared the results with published studies of the nests of other birds and a mammal (common mole, *Talpa europaea*), and also with communities of mites inhabiting the soil. The field research was conducted in the strict nature reserve of the Białowieża National Park, a near-primeval European temperate forest. In 2019, immediately after the breeding period, 69 wood warbler nests and 439 soil samples were collected. Analyses revealed assemblages of Uropodina mites inhabiting the nests that consisted of 14 species, mostly common soil species. Only five species of oribatid mites from superfamily Crotonioidea were present in the nest material. Analyzed nests had a high percentage of tree leaves and grass blades, whereas moss was the least frequent component of the nest material. The Uropodina mites were more abundant in the nests that had greater amounts of grass blades, but similar relationships were insignificant for the nests with varying amounts of tree leaves or moss. The assemblages of Uropodina mites inhabiting wood warbler nests were very similar to those found in soil and nests of the common mole, but they lacked typical nest-dwelling species of Uropodina (i.e., specialized nidicoles).

## Introduction

The research initiated by Nordberg (1936) into relationships between birds and different invertebrates inhabiting their nests has been successfully continued since then. However, studies rarely refer to multiple groups of invertebrates (Tryjanowski et al. 2001) and are often confined only to one taxonomic group. Most published studies have so far focused on mites (Acari) from the order Mesostigmata, which frequently occur in bird nests. This taxon includes many species that appear to prefer bird nest habitats, including both obligatory bird parasites and facultative species, which are not directly associated with the host (see, e.g., Błoszyk et al. 2016).

Since the mid 1980′s several studies have focused on mites from the suborder Uropodina (Acari: Mesostigmata) inhabiting bird nests, but all involved only arboreal or above-ground nest locations, and no study has considered the numerous bird species that nest on the ground; these include studies from Poland (Błoszyk and Olszanowski 1985, 1986; Gwiazdowicz 2003; Gwiazdowicz et al. 1999, 2000, 2005, 2006; Gwiazdowicz and Mizera 2002; Błoszyk et al. 2005, 2006, Bajerlein et al. 2006; Błoszyk and Gwiazdowicz 2006) and Slovakia (Fend’a and Pinowski 1997; Krumpal et al. 1997; Fend’a et al. 1998; Kristofik et al. 2001, 2005, 2007; Mašán 2001, Mašán and Kristofik 1993, 1995; Mašán and Orszagova 1995; Fend’a and Schnierová 2004).

The results of the research into Uropodina mites inhabiting bird nests show that some mites strongly prefer this type of microhabitat, i.e., the (specialist) nidicoles (Lexico 2021), which are not parasites but they reside in bird nests due to the specific microclimatic conditions or maybe some food resources (such as particular nematodes or fungi) (see, e.g., Błoszyk and Olszanowski 1985, 1986; Błoszyk et al. 2006, 2011; Napierała and Błoszyk 2013; Błoszyk et al. 2016). Such species have been found so far in the arboreal nests of several species of bird and a subterranean mammal, the common mole (*Talpa europaea* L.). Typical species of uropodine which are specialist nidicoles inhabiting bird nests are *Leiodinychus orbicularis* (CL Koch), *Apionoseius infirmus* (Berlese), *Nenteria pandioni* (Wiśniewski and Hirschmann), and *Nenteria floralis* (Karg). The nests of small mammals, especially those of the common mole, are frequently inhabited by *Phaulodiaspis borealis* (Sellnick), *Phaulodiaspis rackei* (Oudemans), and *Uroseius hunzikeri* (Schweizer) (Błoszyk et al. 2006; Napierała and Błoszyk 2013).

Importantly, the species outlined above are not only characteristic of bird and mole nests, but they are also dominants (over 15.1% of entire assemblage) in mite communities of these microhabitats (Błoszyk et al. 2006; Napierała and Błoszyk 2013). In the current study, we undertake the first analysis of the species composition of Uropodina inhabiting bird nests built on the ground, in this case by wood warblers [*Phylloscopus sibilatrix* (Bechstein)], a migratory songbird that breeds in temperate Eurasian forests (Cramp 1992). The nests of wood warblers are dome-shaped, built of woven grass, leaves and moss, lined with fine grasses and/or animal hair, and placed among leaf litter or sparse vegetation on the ground (Wesołowski 1985; Cramp 1992). Uropodina mites are part of a larger nest-dwelling community of invertebrates inhabiting wood warbler nests (e.g., ants; Maziarz et al. 2018), but have not previously been documented. The study is the first investigation of the species composition of Uropodina mite and Crotonioidea (Acari: Oribatida) inhabiting wood warbler nests.

Our primary hypothesis was that the abundance of the mite assemblage would depend on the construction of wood warbler nests, including the composition of nesting material, and assuming differences in habitat requirements of different mite species (Błoszyk 1999; Błoszyk et al. 2003; Napierała and Błoszyk 2013). To test this hypothesis, we first assess the main characteristics of the wood warbler nests, and then we analyze the mite numbers in relation to the type of the nest material used. Additionally, we review and compare the fauna of mite assemblages inhabiting the nests of other birds and mammals, derived from the literature, including nests consisting of various types of materials and locations (underground, in nest boxes, on tree branches, etc.). Our initial assumption was that the species composition of mite assemblages inhabiting the wood warbler nests would be similar to those in nests of mammals nesting underground, and different from the nests of other birds that build their nests in more elevated situations.

The results are the first to compare the species composition of Uropodina and Crotonioidea in the nests of wood warblers and other birds, as well as the common mole, and also those found in soil samples, including new data from the near-primeval stands of the iconic Białowieża Forest. We discuss the results in the context of nests as microhabitats for mites.

## Material and methods

### Study area

The research was conducted in the Białowieża Forest, in eastern Poland, which is an exemplar forest area of the temperate climate zone in Europe (Jaroszewicz et al. 2019). We collected the majority of the wood warbler nests (n = 61) from deciduous (*Tilio-Carpinetum*) or mixed-coniferous (*Pino-Quercetum*) forest tree stands in the strictly protected part of Białowieża National Park (henceforth BNP; coordinates of Białowieża village: 52°42′N, 23°52′E). The remaining eight nests come from the tree stands of commercial forests adjacent to BNP.

The strictly protected stands within BNP are a relic of the mixed-coniferous and deciduous temperate forests, which once covered European lowlands, before they were transformed by humans. The pristine features of the old-growth stands in BNP include a multispecies and multistoried structure consisting of trees at different age (up to a few hundred years old), and large abundance of standing and fallen dead trees, as well as with high species diversity (Tomiałojć et al. 1984; Tomiałojć and Wesołowski 2004). The deciduous stands (*Tilio-Carpinetum*), which dominate in BNP, are mainly formed by hornbeams (*Carpinus betulus* L.), limes (*Tilia cordata* Miller), and oaks (*Quercus robur* L.), with admixture of other tree species, including maples (*Acer platanoides* L.) and spruce trees [*Picea abies* (L.) H. Karst.] (Faliński 1986). The dominant tree species in the mixed-coniferous (*Pino-Quercetum*) stands are spruce, pine (*Pinus sylvestris* L.) and oak, with less abundant birches (*Betula* spp.), but there is also an increasing number of hornbeam and lime in recent decades (e.g., Wesołowski et al. 2015).

The adjacent tree stands of the commercial forests have a more uniform structure than those in BNP, with fewer tree species and often younger, with relatively sparse dead wood. The most common tree species were hornbeams and oaks, with less frequent birches, aspens (*Populus tremula* L.), and spruce trees.

### Data collection

We extracted mite specimens from 69 wood warbler nests which were collected in 2019. The nests were collected a median five days following the departure of warbler nestlings (n = 25 nests) or failure of the breeding attempt (mostly due to predation: Wesołowski and Maziarz 2009; Maziarz et al. 2019), if the nest structure remained intact (n = 43). One nest was completed but no eggs were laid within it. Nest collection took place between 27th May and 14th July 2019. Nests were collected in one piece and each placed into a sealed plastic bag with a label describing the nest identity and collection date. The nests were stored in a refrigerator for several days and then transferred to a Berlese-Tullgren funnel for invertebrate extraction.

The process of mite extraction lasted from 56 to 82 h for each nest. The specimens were collected in 100 ml plastic bottles filled with c. 30 ml of 80% ethanol, labelled and placed under each funnel. The mite specimens were sorted out and identified using a stereoscopic microscope (Olympus BX51). The identification of the extracted species was carried out by the last author. The nests were then placed back into their original bags until the examination of nesting material composition.

During the nest material examination, each nest was carefully cleaned by disposing of soil and other debris with tweezers. Next, we divided the material into three major categories: (i) the external layer composed mainly of dead tree leaves, phloem, twigs and individual blades of grass, (ii) the internal layer of the nest walls and the nest cup (which held the birds’ eggs or nestlings) comprised mainly of blades and stems of grass and occasional animal hair, and (iii) the abundant moss extracted from both layers, but mainly from the external one. We placed each part of the nest in a separate container, dried the material for 72 h at 70 °C, and then weighed them separately with a precision of 0.001 g. This procedure was repeated for each nest.

The literature review yielded data for the comparative analysis of mite assemblages inhabiting other bird nests from over 800 open nests and nest boxes of 25 species, with a further 40 samples from nests of the common mole, and 439 soil samples collected in BNP. The material analyzed in this study has been deposited in the Natural History Collections (NHC) at the Faculty of Biology at Adam Mickiewicz University in Poznań. Some datasets used in this study were previously published in earlier studies (Błoszyk et al. 2006, 2011; Napierała and Błoszyk 2013; Błoszyk et al. 2016; Napierała et al. 2016).

### Data analysis

The zoocenological analysis of Uropodina and Crotonioidea assemblages is based on the indices of their dominance and frequency. The following classes were used (Błoszyk 1999):

Dominance: D5, eudominants (> 30%); D4, dominants (15.1–30.0%); D3, subdominants (7.1–15.0%); D2, residents (3.0–7.0%); and D1, subresidents (< 3%).

Frequency: F5, euconstants (> 50%); F4, constants (30.1–50%); F3, subconstants (15.1–30.0%); F2, accessory species (5.0–15.0%); and F1, accidents (< 5%).

The assemblage similarity (S) of species composition for Uropodina mites inhabiting wood warbler nests, and those found in nests of other bird species and the common mole, was calculated by means of the Marczewski-Steinhaus species similarity index: MS = c/(a + b − c), where c is the number of species present in both compared communities, and a and b are the total numbers of species in each assemblage (Magurran 2004). Full joining analysis, which uses the most distant neighbors was used to prepare the dendrogram.

To characterize the composition of the wood warbler nests, we assessed the percentages of the three main nest components (tree leaves, grass blades and mosses) in each nest, expressed as the percentage of the total nest mass. The total mass of a nest was a sum of the tree leaves, grass blades and mosses, thus the percentages of the three components summed to 100%.

To assess the quantitative differences in the Uropodina assemblage in relation to the composition of the wood warbler nests, we used Zero-Inflated Negative Binomial models in R (package *pscl*; Zeileis et al. 2008; Jackman 2020; R Core Team 2020). The models accounted for the large number of zero cases, where Uropodina were absent in the warbler nests. Zero-Inflated Negative Binomial models evaluated the statistical significance of the changes in the abundance and occurrence of Uropodina mites found in the nests built of different amounts (mass) of tree leaves, grass blades and moss. Due to the low abundance of Crotonioidea in the analyzed material, we could not statistically ascertain whether certain types of nesting material had any impact on the abundance of these mites.

Additionally, the significance of any differences in the Uropodina abundance between the successful and failed nests was calculated with the U Mann–Whitney test in R.

## Results

### Uropodina and Crotonioidea in nests of the wood warbler

The assemblage of Uropodina mites inhabiting the wood warbler nests contained 595 specimens in total, including 14 species (Table 1). The most common were *Oodinychus ovalis*, *Trachytes aegrota* and *Urodiaspis tecta*, which constituted from 11 to 48% of the whole assemblage and occurred in 30–63% of all of the wood warbler nests examined (Table 1). The remaining 11 species were less numerous and occurred in fewer nests. Only five species of Crotonioidea were found in the wood warbler nests, including *Heminothrus peltifer* which was the most abundant and most frequent (Table 2).

**Table 1:** Species composition and assemblage structure of Uropodina mites in nests of the wood warbler (with developmental forms)

Species	Total	F	M	D	P	L	D%	F%
*Oodinychus ovalis* (CL Koch)	284	75	98	72	27	12	47.7	62.7
*Trachytes aegrota* (CL Koch)	95		71	16	6	2	16.0	29.9
*Urodiaspis tecta* (Kramer)	64		41	15	7	1	10.8	37.3
*Neodiscopoma splendida* (Kramer)	41	3	9	23	6		6.9	6.0
*Olodiscus minima* (Kramer)	33	27		4		2	5.6	19.4
*Dinychus arcuatus* (Trägårdh)	29	8	15	6			4.9	16.4
*Dinychus perforatus* Kramer	26	12	7	7			4.4	14.9
*Trachytes pauperior* (Berlese)	16	8		3		5	2.7	13.4
*Trematurella elegans* (Kramer)	2	1			1		0.3	3.0
*Uroplitella paradoxa* (Canestrini et Berlese)	1	1					0.2	1.5
*Uropoda orbicularis* (Müller)	1			1			0.2	1.5
*Dinychus woelkei* Hirschmann et Zirngiebl-Nicol	1		1				0.2	1.5
*Urodiaspis pannonica* Willmann	1	1					0.2	1.5
*Uroobovella obovata* (Canestrini et Berlese)	1			1			0.2	1.5
Total	595	136	242	148	47	22	100.0	

*F* female; *M* male; *D* deutonymph; *P* protonymph; *L* larva; *D%* dominance; *F%* frequency

**Table 2:** Crotonioidea species found in nests of the wood warbler

Species	N	D%	F%
*Heminothrus peltifer* (CL Koch)	79	51.6	62.7
*Nothrus palustris* CL Koch	40	26.1	22.4
*Nothrus anauniensis* Canestrini et Fanzago	32	20.9	17.9
*Heminothrus longisetosus* Willmann	1	0.7	1.5
*Camisia solhoeyi* Colloff	1	0.7	1.5
Total	153	100.0	

*N* number of specimens; *D%* dominance; *F%* frequency of occurrence in samples

### Composition of the wood warbler nests

**Fig. 1:**
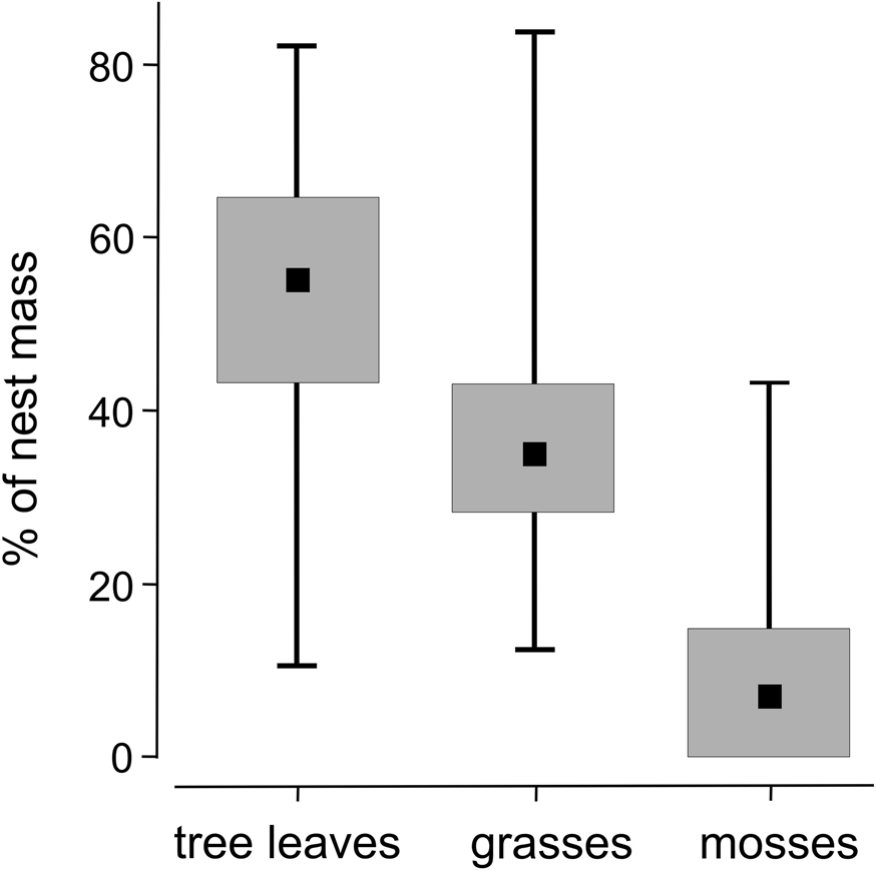
Percentage of total nest mass for the three main nest material components (tree leaves, grass blades and mosses) in the wood warbler nests. Shown are medians (black squares), 25–75 percentiles (grey rectangles) and data ranges (whiskers)

The wood warbler nests consisted mainly of tree leaves, grass blades and mosses, but the percentage mass of each material varied between the nests (Fig. 1). Most of the nests were composed of tree leaves, which constituted 55.2% (median) of the total mass of a wood warbler nest, and grass blades, 35.0% (median). Mosses were the least frequent component of the nest material. As many as 38.8% of all nests contained no mosses at all or trace amounts. The median share of mosses was only 6.9% of the total mass of a nest (Fig. 1).

### The abundance of mites in relation to nest composition and outcome of nesting

**Fig. 2:**
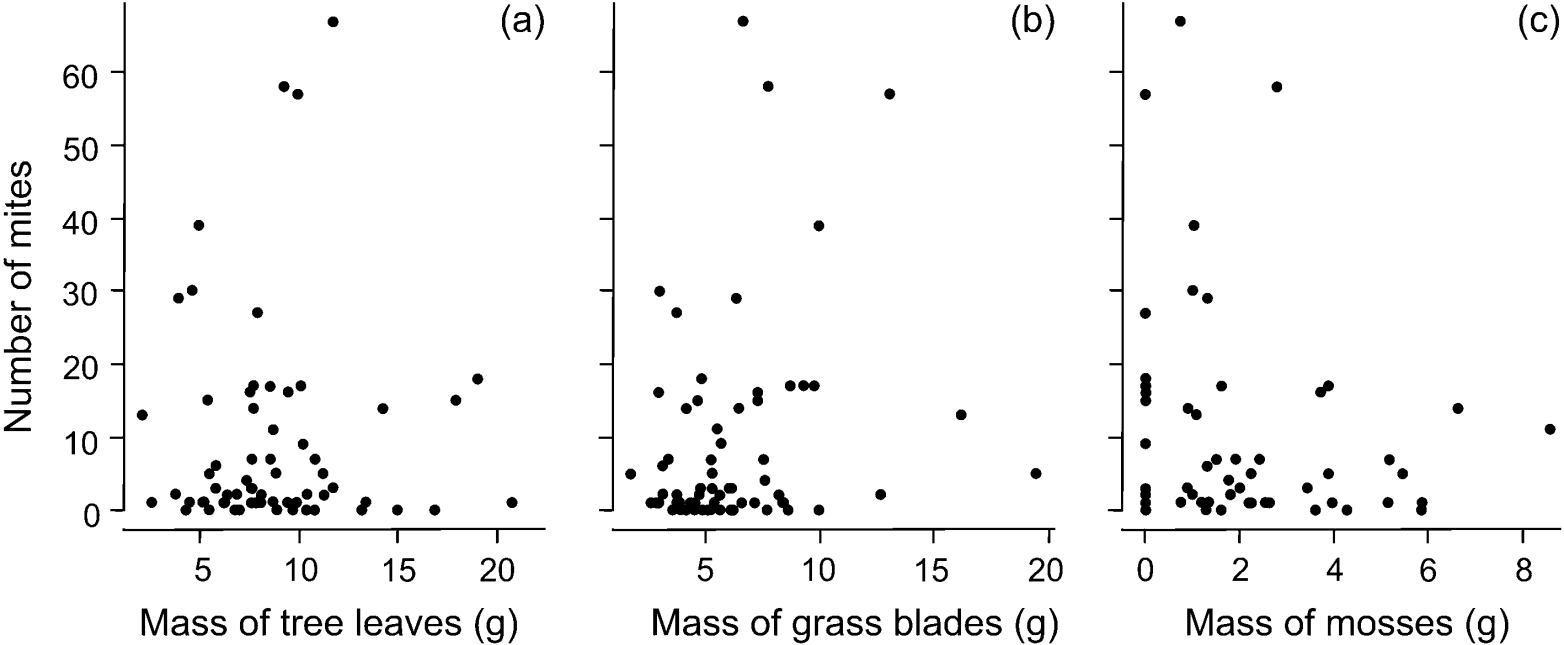
Changes in the abundance of Uropodina mites found in the wood warbler nests, in relation to the mass (g) of the three main nest material components: tree leaves (**a**), grass blades (**b**) and mosses (**c**)

The abundance of Uropodina increased notably with the increasing mass of grass blades in the wood warbler nests (Fig. 2b, Table 3); this relationship was significant for the nests in which mites were found. However, the likelihood of the occurrence of Uropodina was unrelated to the mass of grass blades. Both the abundance and the occurrence of uropodine mites were unrelated to differing amounts of tree leaves or mosses in the nests (Fig. 2a, c, Table 3).

**Table 3:** Results of Zero-Inflated Negative Binomial models showing the relationship between the number of Uropodina mite specimens (response variables) and the mass (g) of tree leaves, grass blades, or mosses (covariates) forming the wood warbler nests in the Białowieża Forest (Poland)

Variable	Tree leaves				Grass blades				Mosses			
	Estimate	SE	z	*p*	Estimate	SE	z	*p*	Estimate	SE	z	*p*
Count model (negbin with log link)												
Intercept	2.162	0.47	4.608	< 0.001	1.318	0.44	2.997	0.003	2.387	0.25	9.633	< 0.001
Mass (g)	0.001	0.05	0.030	0.98	0.129	0.07	1.974	0.048	−0.081	0.09	−0.921	0.36
Log (theta)	−0.791	0.18	−4.364	< 0.001	−0.723	0.18	−3.922	< 0.001	−0.613	0.21	−2.914	0.004
Zero-inflation model (binomial with logit link)												
Intercept	−5.224	1216.52	−0.004	1.0	−6.041	232.09	−0.026	0.98	−1.443	0.83	−1.744	0.081
Mass (g)	−2.535	560.64	−0.005	1.0	−1.522	95.86	−0.016	0.99	−11.981	149.24	−0.080	0.94

Tree leaves: theta = 0.45, log-likelihood = −205.9; grass blades: theta = 0.49, log-likelihood = −203.7; moss: theta = 0.54, log-likelihood = −204.7

The abundance of Uropodina was also similar between the successful (with at least one chick fledged) and failed nests of wood warblers; the respective mean (± SD) number of mite specimens amounted to 5.9 ± 8.1 (n = 25 nests) in successful nests and 9.5 ± 15.9 (n = 43 nests) in failed nests (Mann–Whitney U test: W = 545.5, *p* = 0.92).

### Species similarity of uropodine mites inhabiting nests of the wood warbler and those found in soil and nests of other birds and mammals

**Fig. 3:**
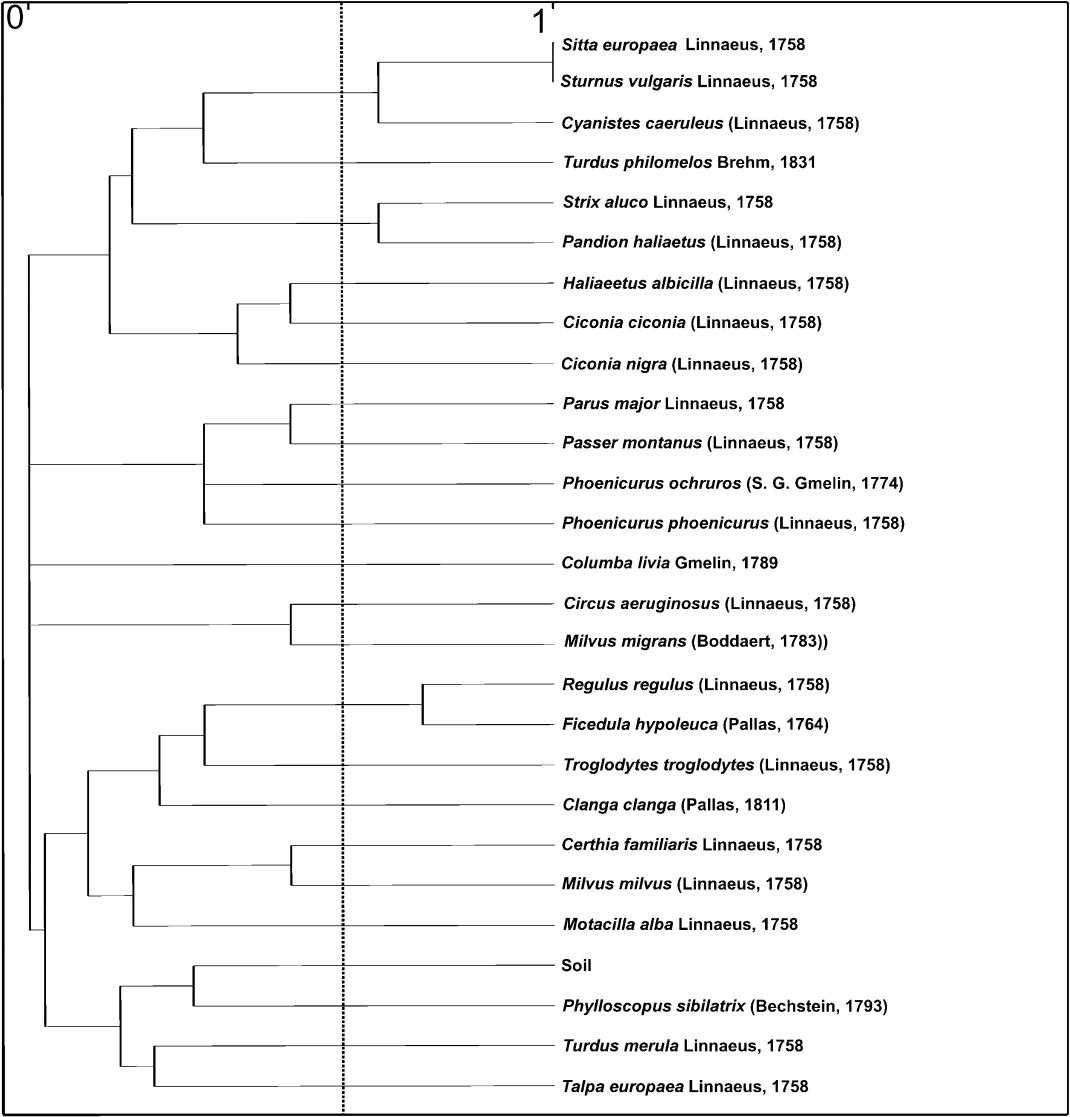
Species similarity (S) of the species composition in Uropodina assemblages inhabiting nests of the wood warbler and nests of 24 other species of birds, the nests of the common mole, and the soil in Białowieża National Park

The extensive material collected for this study allowed comparison between the species composition of Uropodina assemblage inhabiting nests of the wood warbler with those living in nests of 24 bird species in Poland, and from nests of the common mole, as well as from soil samples from BNP (Fig. 3, Table 4).

**Table 4:** List of uropodine mites found in the investigated nests of birds and common mole

		*Talpa europaea* L. European mole	*Ciconia ciconia* (L.) white stork	*Phylloscopus sibilatrix* (Bechstein) wood warbler	*Ciconia nigra* (L.) black stork	*Haliaeetus albicilla* (L.) white-tailed eagle	*Turdus merula* L. common blackbird	*Clanga clanga* (Pallas) greater spotted eagle	*Troglodytes troglodytes* (L.) Eurasian wren	*Turdus philomelos* Brehm song thrush	*Motacilla alba* L. white wagtail	*Ficedula hypoleuca* (Pallas) European pied flycatcher	*Pandion haliaetus* (L.) osprey	*Cyanistes caeruleus* (L.) Eurasian blue tit	*Regulus regulus* (L.) goldcrest	*Sturnus vulgaris* L. common starling	*Passer montanus* (L.) Eurasian tree sparrow	*Sitta europaea* L. Eurasian nuthatch	*Phoenicurus phoenicurus* (L.) common redstart	*Milvus milvus* (L.) red kite	*Strix aluco* L. tawny owl	*Milvus migrans* (Boddaert) black kite	*Phoenicurus ochruros* (SG Gmelin) black redstart	*Parus major* L. great tit	*Certhia familiaris* L. Eurasian treecreeper	*Columba livia* Gmelin common pigeon	*Circus aeruginosus* (L.) western marsh harrier	Σ
Species	Soil																											
Nest use (A, perennial nests; B, 1-year nests; C, nest-boxes)	–	A	A	B	A	A	B	A	B	B	B	C	A	C	B	C	B	B	B	A	B	A	C	C	B	B	B	
Nest sites (G, ground; wG, wet ground; S, soil; T, tree; H, hollow; B, buildings; NB, nest-box; P, pole)	–	S	P	G	(T)*	T	(T)	(T)	(T)	(T)	H, (B, NB)	NB	T	NB	(T)	NB	NB	NB	NB	T	NB	(T)	B	NB	H	B	wG	
Month of collection	–		VI	V, VI, VII	VI, VII	V, VI	X, XII	VI, VII	VI, XI	V, X	VIII	VI, X	VI	VI, X	VI	VI, X	X	X	X	VI	VI	V, VI	V	VI, X	VI	II, VII, X, XI	VII	
*Leiodinychus orbicularis* (CL Koch)	+	+	+		+	+	+			+		+		+		+	+	+	+				+	+				14
*Trachytes aegrota* (CL Koch)	+	+		+	+		+	+	+		+	+			+					+					+			12
*Oodinychus ovalis* (CL Koch)	+	+	+	+	+	+	+			+		+	+		+						+							12
*Apionoseius infirmus* (Berlese)	+		+		+	+		+		+			+	+		+		+			+							11
*Uropoda orbicularis* (Müller)		+	+	+	+	+					+											+					+	8
*Dinychus perforatus* Kramer	+	+		+				+	+			+			+													7
*Uroobovella pyriformis* (Berlese)	+		+		+	+			+					+														6
*Nenteria pandioni* Wiśniewski et Hirschmann			+			+							+							+		+						5
*Olodiscus minima* (Kramer)	+	+	+	+			+																					5
*Oodinychus karawajewi* (Berlese)	+	+	+							+	+																	5
*Dinychus arcuatus* (Trägårdh)	+			+		+																	+					4
*Nenteria breviunguiculata* (Willmann)		+	+		+																							3
*Uroobovella obovata* (Canestrini et Berlese)		+	+	+				+																				4
*Urodiaspis tecta* (Kramer)	+			+			+																					3
*Polyaspis patavinus* Berlese		+																	+									2
*Uroseius hunzikeri* Schweizer		+					+																					2
*Uroobovella marginata* (CL Koch)			+			+																						2
*Phaulodiaspis rackei* (Oudemans)		+																										1*
*Phaulodiaspis borealis* Sellnick		+																										1*
*Trachytes pauperior* (Berlese)	+			+																								2
*Trematurella elegans* (Kramer)	+			+																								2
*Neodiscopoma splendida* (Kramer)	+			+																								2
*Uroplitella paradoxa* (Canestrini et Berlese)				+																								1
*Dinychus woelkei* Hirschmann et Z.-Nicol	+			+																								2
*Olodiscus misella* (Berlese)	+	+																										2
*Pseudouropoda calcarata* (Hirschmann et Z.-Nicol)		+																										1*
*Dinychus carinatus* (Berlese)	+	+																										2
*Dinychus inermis* (CL Koch)		+																										1*
*Discourella modesta* (Leonardi)	+	+																										2
*Pseudouropoda tuberosa* (Hirschmann et Z.-Nicol)							+																					1
*Urodiaspis pannonica* (Willmann)	+			+			+																					3
*Uroobovella flagelliger* (Berlese)			+																									1
*Pseudouropoda* sp.	+		+																									2
*Polyaspinus cylindricus* (Berlese)																										+		1
*Nenteria floralis* Karg								+																				1
*Iphiduropoda penicillata* (Hirschmann et Z.-Nicol)																	+											1
*Pseudouropoda structura* (Hirschmann et Z.-Nicol)										+																		1
*Discourella cordieri* (Berlese)											+																	1
*Uropoda hamulifera* Michael								+																				1
*Cilliba sellnicki* (Hirschmann et Z.-Nicol)									+																			1
*Uroobovella fimicola* (Berlese)					+																							1
*Uroobovella pulchella* (Berlese)	+																											1**
*Uroobovella ipidis* Hirschmann et Z.-Nicol,	+																											1**
*Uroobovella baloghi* Hirschmann et Z.-Nicol	+																											1**
*Polyaspis sansonei* Berlese	+																											1**
*Cilliba insularis* Willmann	+																											1**
*Dinychus septentrionalis* (Trägårdh)	+																											1**
*Trichouropoda polytricha* (Vitzthum)	+																											1**
*Uropoda fumicola* Hirschmann et Z.-Nicol	+																											1**
*Olodiscus kargi* (Hirschamann et Z.-Nicol)	+																											1**
*Trichouropoda bipilis* (Vitzthum)	+																											1**
*Oplitis* sp.	+																											1**
*Uropolyaspis hamulifera* Berlese	+																											1**
*Discourella (?) baloghi* Hirschmann et Z.-Nicol	+																											1**
Number of Uropodina species in nest	32	18	13	12	8	8	8	6	5	5	4	4	3	3	3	2	2	2	2	2	2	2	2	1	1	1	1	
Number of samples	439	40	38	69	105	30	43	20	5	20	10	79	10	25	3	40	15	6	4	6	3	4	4	78	2	30	4	
Number of nests		40	30	69	34	12	43	10	5	20	10	79	5	25	3	40	15	6	4	2	1	2	4	78	2	30	2	
Lack of nidicole species				X					X		X				X										X	X	X	

*Only in mole nests

**Only in soil

The compared material contained 54 species of uropodine mites, of which 36 species occurred in the nests of birds, only four species in the nests of the common mole, and 13 species in the soil samples (Table 4). Moreover, 11 species of Uropodina were found in the nests of only a single bird species. The number of mite species inhabiting the analyzed nests varied between species, ranging from 18 in nests of the common mole to only one in nests of the great tit (*Parus major* L.), Eurasian treecreeper (*Certhia familiaris* L.), feral rock pigeon (*Columba livia* JF Gmelin) and western marsh harrier (*Circus aeruginosus* L.). Only four species of Uropodina occurred in the nests of ten or more bird species. The nests of seven bird species contained no specialist nidicolous species of Uropodina.

The analysis of similarity (S) of the bird nests and those of the common mole, and the communities in the soil samples, revealed that the assemblages of uropodine mites inhabiting wood warbler nests were most similar to those found in the soil samples, rather than to communities in the nests of any other bird (Fig. 3). However, compared to other birds, the species composition of the Uropodina communities inhabiting the nests of the common mole and common blackbird (*Turdus merula* L.) were more similar to that of the wood warbler nests.

## Discussion

The analysis of the Uropodina assemblage revealed that all species found in nests of the wood warbler are common mite species of soil fauna, characteristic of Central Europe. The most dominant species in wood warbler nests was *O. ovalis*, which is also one of the most common Uropodina species in Poland, both in soil and dead wood. This phoretic species is carried by beetles and centipedes (Błoszyk 1999; Błoszyk et al. 2003; Napierała et al. 2015). Also quite abundant were *T. aegrota* and *U. tecta*, two species that frequently occur in forest litter and soils, especially in deciduous forests (Błoszyk 1999; Mašán 2001; Błoszyk et al. 2003). Another group characterized by high abundance and frequent occurrence comprises *Neodiscopomma splendida*, *Olodiscus minima*, *Dinychus arcuatus*, *D. perforatus*, and *T. pauperior*.

The rarer mites in the wood warbler nests included *Trematurella elegans*, *Urooplitella paradoxa*, *Uropoda orbicularis*, *U. pannonica*, *Dinychus* w*oelkei*, and *Uroobovella* sp. The first of these, which is often associated with old forests, has a widespread occurrence in Poland, except for areas with high mountains (Błoszyk 1999; Błoszyk et al. 2018). *Urooplitella paradoxa* is a rare species that occurs in low abundance in Poland (the IUCN Red List category VU) (Napierała et al. 2018). *Uropoda orbicularis* is a common species with a wide range of occurrence, whose deutonymphs are carried by beetles (Bajerlein and Błoszyk 2004; Napierała et al. 2018). *Dinychus woelkei* is a species usually found in dead wood (Błoszyk 1999; Mašán 2001).

Consequently, the core of mite assemblages in wood warbler nests consists mainly of soil species also found in BNP. However, differences in the geographical range of occurrence of these mites in Poland means that the species composition of Uropodina assemblage in wood warbler nests could slightly differ in each region of the country. For example, material obtained from a large series of nests collected in Wielkopolska (Greater Poland) contained no specimens of *N. splendida* because this species does not occur in this region (Błoszyk, unpubl. data).

As for Crotonioidea in open bird nests and nest boxes in Poland, Błoszyk and Olszanowski (1985) were the first to describe the species composition of mites from this group. The material from the nests and nest boxes of 11 bird species of birds contained six mite species from this superfamily. In the current study, four out of five species of crotonioids that were found in wood warbler nests were also recorded in other bird nests in earlier studies (Błoszyk and Olszanowski 1985; Olszanowski 1996). The most frequent species found was *H. peltifer*, which is the most common eurytopic species from this group in Poland, with widespread range (Olszanowski 1996). Also, *Nothrus palustris* was abundant, and is a common species in Poland that prefers damp habitats (Olszanowski 1996). The wood warbler material also contained many specimens of *Nothrus anauniensis*, which is a slightly less common species of various habitats, especially scrub, city parks and in grass growing on limestone (Olszanowski 1996). The least abundant species was *Heminothrus longisetosus*, which is rare in Poland with a strong preference for open habitats, such as meadows, and nests of mammals and birds. The only species from the wood warbler nests that has previously never been found in any bird nest was *Camisia solhoeyi* (Olszanowski 1996). This species is very rare and has been recorded only from southern Poland (Olszanowski 1996).

The results show that the nesting material used by the host bird has a significant impact on the abundance of uropodine mites, whereas the nesting outcome (fledged vs. failed nests) was unimportant. Błoszyk (1985) showed that nests of the common mole that are built of grass, or layers of grass and leaves, contain far more Uropodina mites than those built only of leaves. A similar situation was observed in the wood warbler nests. This suggests that grasses used as a nesting material probably creates favorable habitat conditions for Uropodina. Notably, the uropodine fauna was the most similar in the nests of species that breed underground and use predominantly grasses (and some tree leaves) in nest building, such as common mole, and/or line the nest with a layer of soil/mud, such as blackbird (Fig. 3).

One of the most evident characteristics of the Uropodina assemblage inhabiting the wood warbler nests is the lack of typical (specialist) nidicoles. Earlier studies that discuss this issue in relation to material from permanent and seasonal nests, as well as from nest boxes, show that in most analyses of bird nests there was always at least one uropodine species regarded as a typical nidicole (Błoszyk et al. 2006; Napierała and Błoszyk 2013). A similar lack of specialist nidicoles as found in the wood warbler nests was observed in only a few other bird species, as shown in some earlier studies (Błoszyk et al. 2006); specialist nidicoles have not been found in nests of the Eurasian wren (*Troglodytes troglodytes* L.), white wagtail (*Motacilla alba* L.), goldcrest (*Regulus regulus* L.), Eurasian treecreeper, feral rock pigeon and western marsh harrier (Table 4). However, for all but two of these bird species only a few nests were examined (usually only one sample). Thus, the lack of specialist nidicoles in these nests may stem from the low number of analyzed nests. Only in the feral rock pigeon and the wood warbler was the number of analyzed nests high enough (30 and 69, respectively) to consider the obtained results as reliable, reflecting the actual species composition of the Uropodina assemblage in these nests.

The analysis of species similarity of the Uropodina assemblage in the nests of the wood warbler and those of the other 24 bird species, as well as the nests of the common mole and in the soil samples from BNP, showed that the assemblage in the wood warbler nests was most similar to those in the soil (Fig. 3). The strong similarity in the species composition between the material from the nests of the wood warbler and from the soil samples can be explained by the fact that this bird builds nests directly on the ground surface. The proximity or use of soil in the nests of common moles and common blackbirds may also reflect their similarity to wood warbler nests.

The absence of specialist nidicoles in the wood warbler nests could result from the nests being collected soon after the birds’ breeding attempts, each of which only lasts for a maximum of approximately 35 days (Cramp 1992). For this reason, the development of a nest assemblage with typical nidicoles in such a short period of time was probably impossible. This is likely why the analyzed communities consisted mainly of soil species, probably brought by the host bird species with the nesting material.

A similar tendency can be observed during succession in areas destroyed by environmental or ecological disasters, for example large fires, industrial heaps and areas of melting glaciers (see, e.g., Michalik 2001; Skubała and Gulvik 2005; Seniczak et al. 2006; Hågvar et al. 2009). Such areas are usually colonized initially by common eurytopic species coming from the adjacent areas, and then gradually an assemblage is formed of particular species that are specific to a given type of environment. However, in order to ascertain whether the lack of specialist nidicoles in the wood warbler nests stems from the method used for collecting the nests (the nest collection period), or whether it is indeed a typical characteristic of Uropodina assemblage inhabiting nests of this bird species, further research is necessary.
